# Comparative effectiveness of finerenone versus steroidal mineralocorticoid receptor antagonists on sepsis-induced cardiomyopathy in patients with diabetic kidney disease: a real-world cohort study

**DOI:** 10.3389/fphar.2026.1837944

**Published:** 2026-07-28

**Authors:** Jheng-Yan Wu, Keng-Wei Lee, Sheng-Chi Huang, Hsuan-Yuan Chang, Yu-Min Lin

**Affiliations:** 1 Department of Nutrition, Chi Mei Medical Center, Tainan, Taiwan; 2 Department of Public Health, College of Medicine, National Cheng Kung University, Tainan, Taiwan; 3 Department of Internal Medicine, Division of Cardiology, Chi Mei Medical Center, Chiali, Tainan, Taiwan; 4 Department of Medical Education, Chi Mei Medical Center, Tainan, Taiwan; 5 Department of Internal Medicine, Division of Hepatogastroenterology, Chi Mei Medical Centre, Tainan, Taiwan; 6 Department of Internal Medicine, Division of Cardiology, Chi Mei Medical Center, Tainan, Taiwan

**Keywords:** Diabetic kidney disease, finerenone, mineralocorticoid receptor antagonists, real-world evidence, sepsis, sepsis-induced cardiomyopathy

## Abstract

**Background:**

Sepsis-induced cardiomyopathy (SICM) is a common but underrecognized complication of sepsis and is associated with adverse short- and long-term outcomes. Patients with diabetic kidney disease (DKD) are particularly vulnerable to sepsis-related cardiac dysfunction, potentially due to chronic inflammation and heightened mineralocorticoid receptor (MR) activation. Whether non-steroidal MRAs are associated with a lower risk of SICM compared with steroidal MRAs remains unknown.

**Methods:**

Using the TriNetX federated electronic health record network, we conducted a retrospective cohort study of adults with DKD and sepsis between 2021 and 2025. Patients initiating finerenone were compared with those initiating steroidal MRAs. Propensity score matching (1:1) was performed to balance baseline characteristics. The primary outcome was SICM, defined by heart failure, cardiogenic shock, pulmonary edema, or left ventricular ejection fraction ≤50%. Secondary outcomes included all-cause mortality and surrogate markers of myocardial injury. Cox proportional hazards models were used to estimate hazard ratios (HRs).

**Results:**

After matching, 1,378 patients were included in each group. Finerenone use was associated with a significantly lower risk of SICM compared with steroidal MRAs (3.3% vs 5.2%; HR 0.59, 95% CI 0.40–0.87). Finerenone was also associated with lower all-cause mortality (HR 0.45, 95% CI 0.31–0.65) and a reduced risk of surrogate myocardial injury outcomes (HR 0.48, 95% CI 0.25–0.90). Results were generally directionally similar across several subgroup analyses, although the association was attenuated among patients not receiving SGLT2 inhibitors.

**Conclusion:**

In this real-world cohort of patients with DKD and sepsis, finerenone use was associated with a lower observed risk of clinically recognized SICM compared with steroidal MRAs. A lower all-cause mortality rate was also observed; however, given the magnitude of this association and the observational design, this finding should be interpreted cautiously and may reflect residual confounding or treatment-selection bias. These findings are hypothesis-generating and require confirmation in prospective studies.

## Introduction

Sepsis represents a major and growing global public-health challenge, with recent estimates indicating an enormous and rising burden of disease worldwide (Global Sepsis Collaborators, 2025). Beyond its high mortality, sepsis frequently leads to acute multi-organ dysfunction, among which cardiovascular involvement is increasingly recognized as a critical determinant of clinical outcomes (Lelubre and Vincent, 2018). Sepsis-induced cardiomyopathy (SICM), characterized by transient myocardial systolic dysfunction, cardiac biomarker elevation, and hemodynamic instability, is a common yet underdiagnosed complication of sepsis (Aissaoui et al., 2025; Sato et al., 2025). Accumulating evidence suggests that the development of SICM is associated with substantially worse short-term and long-term outcomes, including higher risks of acute heart failure, cardiogenic shock, prolonged intensive care unit stay, and increased mortality (Lin et al., 2022; Hollenberg, 2025; Hasegawa et al., 2023). Despite its prognostic significance, effective strategies for the prevention or mitigation of SICM remain limited.

Patients with diabetic kidney disease (DKD) experience disproportionately high morbidity and mortality from sepsis (Sarnak and Jaber, 2000; Jiang and Cheng, 2022). DKD is characterized by persistent systemic inflammation, oxidative stress, and heightened mineralocorticoid receptor (MR) activation, which may exacerbate myocardial vulnerability during acute infectious stress (Rangel, 2025; Matoba et al., 2019; Mende et al., 2023). These intertwined cardiorenal and immune abnormalities may render patients with DKD particularly susceptible to SICM and subsequent adverse cardiovascular outcomes.

Mineralocorticoid receptor antagonists (MRAs) are widely used in patients with DKD for their established cardiovascular and renal benefits (Barrera-Chimal et al., 2022; Harrington et al., 2025). Non-steroidal MRAs such as finerenone differ pharmacologically from conventional steroidal MRAs by offering greater receptor selectivity and a more favorable tissue distribution (Harrington et al., 2025). These properties may be clinically relevant in the context of sepsis, a condition characterized by profound inflammatory and hemodynamic stress (Belden et al., 2017; Font et al., 2020). Although finerenone has demonstrated cardiovascular and renal benefits in DKD (Pitt et al., 2021), whether its use is associated with a lower risk of SICM compared with steroidal MRAs remains unknown.

Accordingly, using a large federated real-world electronic health record network (TriNetX), we conducted a comparative effectiveness study to evaluate whether finerenone use, compared with steroidal MRAs, was associated with a lower risk of SICM in patients with DKD. Secondary outcomes included surrogate markers of myocardial injury and dysfunction, and all-cause mortality. This study aimed to provide real-world evidence on the potential role of non-steroidal MRAs in mitigating sepsis-related cardiac complications in a high-risk population.

## Methods

### Data source

This retrospective cohort study utilized data from the TriNetX research platform, which aggregates electronic health records from approximately 160 million patients across 170 healthcare organizations worldwide. The platform provides access to aggregated and de identified data only, including information on clinical diagnoses, laboratory measurements, medical procedures, medication use, and genomic features. Investigators are unable to access protected health information or identify individual participants. Because the database contains no direct personal identifiers and does not allow interaction with patients, review and approval by the Western Institutional Review Board were not required. The study was designed, conducted, and reported in accordance with the Strengthening the Reporting of Observational Studies in Epidemiology guidelines (Cuschieri, 2019).

## Ethical statement

This study was conducted using de-identified data from the TriNetX research network. Investigators had no access to protected health information and no direct patient contact was involved. In accordance with applicable regulations, institutional review board approval and informed consent were not required.

### Study design

We identified adults aged 18 years or older with documented diagnoses of DKD and sepsis between January 1, 2021, and November 30, 2025. DKD and sepsis were ascertained using ICD-10-CM codes N18, R80, and E11, urine albumin to creatinine ratio values of at least 30, and ICD-10-CM codes A00 to B99, respectively. Eligible individuals were categorized into two exposure groups. Patients with at least one prescription for non-steroidal MRAs or steroidal MRAs within the 12 months preceding the diagnosis of sepsis were assigned to the corresponding exposure groups. To ensure that MRA exposure preceded the septic event, only prescriptions recorded before the index sepsis diagnosis were considered. Patients with documented DKD within the same 12-month pre-index period were eligible for inclusion. The index date was defined as the date of sepsis diagnosis, and follow-up for outcomes began on the following day.

Detailed variable definitions and coding algorithms are provided in Supplementary Table S1.

### Covariates and propensity score matching

After the study cohorts, index dates, outcomes, and potential confounders were defined, baseline covariates were constructed using data from the 12 months prior to the index date. Propensity scores were estimated using logistic regression to model the likelihood of receiving the study treatment on the basis of pre exposure characteristics. Patients were matched in a one to one ratio using a greedy nearest neighbor algorithm without replacement, applying a caliper width equal to one-tenth of the pooled standard deviation of the logit transformed propensity score. Covariate balance between groups was considered acceptable when standardized mean differences were below 0.1, consistent with prior recommendations (Haukoos and Lewis, 2015).

Baseline covariates included in the propensity score matching (PSM) were selected based on prior literature to account for demographic characteristics, comorbid conditions, medication use, and relevant laboratory and physiological parameters that may influence treatment allocation and clinical outcomes. Age was summarized as mean with standard deviation. Sex was reported as the number and percentage of female and male patients. Race was categorized as White, Black or African American, and Asian. Comorbidities were presented as counts and percentages and included dyslipidemia, obesity and overweight, nicotine dependence, atrial fibrillation or flutter, other sepsis, and type 2 diabetes mellitus with kidney, ophthalmic, neurological, or circulatory complications.

Medication exposure was also incorporated into the matching procedure and was summarized as the number and percentage of patients receiving angiotensin converting enzyme inhibitors, angiotensin receptor blockers, beta blockers, metformin, and sodium glucose cotransporter 2 inhibitors. Laboratory and clinical variables were additionally included. C reactive protein and lactate levels were reported as mean with standard deviation, with lactate further categorized according to a threshold of at least 2 mmol/L. Estimated glomerular filtration rate (eGFR) was summarized as mean with standard deviation and dichotomized using a cutoff of less than 45 mL/min/1.73 m^2^. Hemoglobin A1c was expressed as mean with standard deviation and the proportion of patients with values of at least 9%. Low density lipoprotein cholesterol was presented as mean with standard deviation and the proportion with levels of at least 160 mg/dL. Body mass index was summarized as mean with standard deviation, with obesity defined as a value of at least 30 kg/m^2^. Full variable definitions and coding criteria are provided in Supplementary Table S2.

### Outcomes and follow-up

The primary endpoint was defined as SICM. Because echocardiographic data are incompletely captured in real-world EHR databases, SICM was not defined by LVEF alone. Instead, SICM was defined as a composite of recorded LVEF ≤50% and clinically coded manifestations of sepsis-related cardiac dysfunction, including heart failure, cardiogenic shock, and pulmonary edema. In TriNetX, absence of a structured LVEF value cannot reliably distinguish between lack of echocardiographic testing, incomplete data capture, or availability of LVEF only in unstructured reports; therefore, formal assessment of LVEF completeness was not feasible.

Secondary outcomes included all-cause mortality and a surrogate outcome. The surrogate outcome was identified using N terminal pro B type natriuretic peptide ≥ 1800 pg/mL, troponin I ≥ 0.5 mg/dL, or left ventricular ejection fraction ≤ 50%.

Outcome follow up began on the day after the index date and continued until the earliest occurrence of the outcome of interest, the last clinical encounter, death, or a maximum of 1 year after cohort entry. Full outcome definitions and corresponding coding criteria are provided in Supplementary Table S3.

### Subgroup analysis

For the primary outcome, subgroup analyses were performed according to the prespecified analytical plan. Patients were stratified by age group (18–64 years and ≥ 65 years), sex, type 2 diabetes status, use of sodium glucose cotransporter two inhibitors, and type of infection, including bacteremia, urinary tract infection, pneumonia, and enterogastritis.

### Sensitivity analysis

To evaluate the robustness of the study findings, skin cancer was included as a negative control outcome. In addition, to mitigate potential time dependent confounding in this observational study, a landmark analysis was performed using a prespecified fixed time point after the index date. Also, the risks of hyperkalemia and acute kidney injury were evaluated to assess the safety profile. An additional analysis was performed using a biomarker-defined myocardial injury, defined as N-terminal pro B-type natriuretic peptide ≥1800 pg/mL or troponin I ≥0.5 ng/mL.

### Statistical analysis

Continuous variables were summarized as mean values with standard deviations, whereas categorical variables were presented as frequencies with corresponding percentages. PSM was applied to balance baseline characteristics between the treatment groups prior to the primary, subgroup, and sensitivity analyses. Hazard ratios (HRs) and 95% confidence intervals (CIs) were estimated using Cox proportional hazards regression models. Time to event outcomes were evaluated using Kaplan Meier survival curves, with between group differences assessed by the log rank test. To assess the potential impact of unmeasured confounding on the observed associations, E values were calculated (VanderWeele and Ding, 2017). All statistical analyses were performed using the TriNetX research platform.

## Results

### Study cohort

**FIGURE 1:**
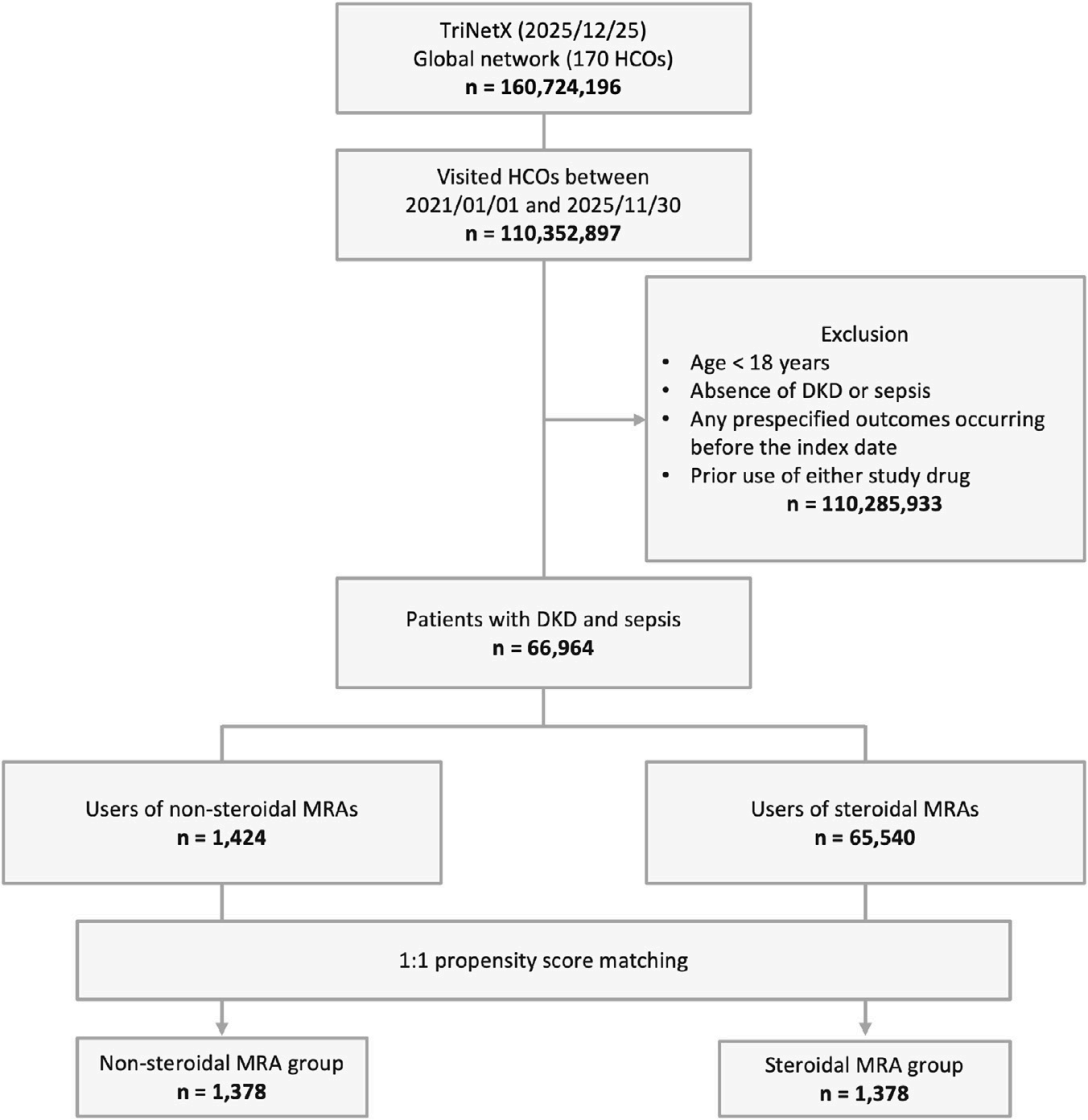
Study cohort process. DKD, diabetic kidney disease; HCOs, healthcare organizations; MRA, mineralocorticoid receptor antagonist.

110,352,897 patients had at least one healthcare encounter between January 1, 2021, and November 30, 2025. After applying the exclusion criteria, 66,964 eligible patients with DKD and sepsis remained. Within this cohort, 1,424 patients were identified as users of non-steroidal MRAs, while 65,540 patients were users of steroidal MRAs. 1:1 PSM was performed, resulting in two well balanced cohorts consisting of 1,378 patients in the non-steroidal MRA group and 1,378 patients in the steroidal MRA group (Figure 1).

### Characteristics of study subjects

Before PSM, substantial imbalances were observed between the non-steroidal MRA group and the steroidal MRA group across multiple baseline characteristics. Patients receiving non-steroidal MRAs were older on average and showed marked differences in sex distribution and race composition. Several comorbidities, particularly type 2 diabetes mellitus with kidney, neurological, ophthalmic, and circulatory complications, as well as dyslipidemia, were more prevalent in the non-steroidal MRA group, with standardized mean differences exceeding 0.3 for multiple variables. Pronounced imbalances were also noted for medication use, especially sodium glucose cotransporter 2 inhibitors and angiotensin receptor blockers, as well as for renal function indicators including estimated glomerular filtration rate and the proportion of patients with eGFR < 45 mL/min/1.73 m^2^ (Table 1).

**TABLE 1:** Baseline characteristics of non-steroidal MRA and steroidal MRA groups before and after propensity score matching.

Variables	Before matching			After matching		
	Non-steroidal MRA group (n = 1,424)	Steroidal MRA group (n = 65,540)	Standardized difference	Non-steroidal MRA group (n = 1,378)	Steroidal MRA group (n = 1,378)	Standardized difference
Age, years						
Mean (SD)	65.9 (11.8)	61.8 (15.2)	0.302	65.7 (11.9)	66.1 (12.8)	0.032
Sex, n (%)						
Female	628 (44.1)	39,709 (60.7)	0.336	615 (44.6)	632 (45.9)	0.025
Male	796 (55.9)	25,714 (39.3)	0.337	763 (55.4)	746 (54.1)	0.025
Race, n (%)						
White	631 (44.3)	36,932 (56.4)	0.244	631 (45.8)	624 (45.3)	0.01
Black or african american	218 (15.3)	12,956 (19.8)	0.118	218 (15.8)	223 (16.2)	0.01
Asian	305 (21.4)	2,127 (3.3)	0.575	259 (18.8)	247 (17.9)	0.022
Comorbidities, n (%)						
Dyslipidemia	949 (66.6)	30,204 (46.1)	0.422	908 (65.9)	896 (65)	0.018
Obesity and overweight	348 (24.4)	17,530 (26.8)	0.054	341 (24.7)	319 (23.1)	0.037
Nicotine dependence	110 (7.7)	6,201 (9.5)	0.062	110 (8)	108 (7.8)	0.005
Atrial fibrillation and flutter	68 (4.8)	4,693 (7.2)	0.101	67 (4.9)	78 (5.7)	0.036
Other sepsis	42 (2.9)	2,703 (4.1)	0.064	42 (3)	37 (2.7)	0.022
Type 2 diabetes mellitus with kidney complications	753 (52.9)	6,988 (10.7)	1.017	708 (51.4)	713 (51.7)	0.007
Type 2 diabetes mellitus with ophthalmic complications	156 (11)	2,329 (3.6)	0.288	150 (10.9)	139 (10.1)	0.026
Type 2 diabetes mellitus with neurological complications	306 (21.5)	6,261 (9.6)	0.334	296 (21.5)	280 (20.3)	0.029
Type 2 diabetes mellitus with circulatory complications	127 (8.9)	2,553 (3.9)	0.206	120 (8.7)	125 (9.1)	0.013
Medications, n (%)						
ACEIs	321 (22.5)	12,322 (18.8)	0.092	315 (22.9)	313 (22.7)	0.003
ARBs	677 (47.5)	18,647 (28.5)	0.4	644 (46.7)	658 (47.8)	0.02
Beta blockers	550 (38.6)	30,018 (45.9)	0.147	538 (39)	526 (38.2)	0.018
Metformin	500 (35.1)	17,935 (27.4)	0.167	484 (35.1)	502 (36.4)	0.027
SGLT2is	731 (51.3)	6,055 (9.3)	1.03	685 (49.7)	692 (50.2)	0.01
C reactive protein, mg/L						
Mean (SD)	37.2 (69.7)	36.7 (56.1)	0.007	37.3 (70.4)	36.2 (51.9)	0.017
Lactate, mmol/L						
Mean (SD)	1.4 (0.6)	1.7 (1.3)	0.23	1.4 (0.6)	1.4 (0.7)	0.109
≥2, n (%)	40 (2.8)	4,761 (7.3)	0.205	40 (2.9)	32 (2.3)	0.036
eGFR, mL/min/1.73m^2^						
Mean (SD)	52.1 (26.1)	71.3 (32.4)	0.656	52.4 (26.2)	58.5 (28.9)	0.223
<45, n (%)	633 (44.5)	15,815 (24.2)	0.438	602 (43.7)	597 (43.3)	0.007
Hemoglobin A1c, %						
Mean (SD)	7.3 (1.6)	6.8 (1.7)	0.263	7.3 (1.6)	7.3 (1.7)	0.004
≥9, n (%)	183 (12.9)	4,800 (7.3)	0.184	177 (12.8)	201 (14.6)	0.051
Cholesterol in LDL, mg/dL						
Mean (SD)	80 (35.6)	89.6 (38.6)	0.258	79.9 (35.1)	81.2 (36.4)	0.037
≥160, n (%)	50 (3.5)	1,783 (2.7)	0.045	45 (3.3)	28 (2)	0.077
Body mass index, kg/m^2^						
Mean (SD)	30.5 (7.1)	32.7 (8.5)	0.281	30.7 (7.2)	30.7 (7.2)	0.002
≥30, n (%)	493 (34.6)	26,164 (40)	0.111	482 (35)	456 (33.1)	0.04

ACEIs, angiotensin-converting enzyme inhibitors; ARBs, angiotensin II, receptor blockers; eGFR, estimated Glomerular filtration rate; LDL, low-density lipoprotein; MRA, mineralocorticoid receptor antagonist; SD, standard deviation; SGLT2is, sodium-glucose cotransporter-2, inhibitors.

After 1:1 PSM, 1,378 patients remained in each treatment group. Baseline characteristics were well balanced between the two groups following matching. Standardized mean differences for all demographic variables, comorbidities, medication use, and laboratory measurements were reduced to below 0.1, indicating adequate covariate balance. Age, sex, and race distributions were comparable between groups after matching. The prevalence of comorbid conditions, including dyslipidemia, obesity and overweight, atrial fibrillation or flutter, and diabetes related complications, was similar across groups. Medication use, including angiotensin converting enzyme inhibitors, angiotensin receptor blockers, beta blockers, metformin, and sodium glucose cotransporter 2 inhibitors, was also well balanced. The steroidal MRA group consisted predominantly of spironolactone users (approximately 95%), with eplerenone accounting for the remaining 5%.

Laboratory and physiological measures, including C reactive protein, lactate levels, estimated glomerular filtration rate, hemoglobin A1c, low density lipoprotein cholesterol, and body mass index, showed comparable distributions between groups after matching, with all standardized mean differences remaining within acceptable limits.

### Primary and secondary outcomes

The primary and secondary outcomes were compared between the non-steroidal MRA group and the steroidal MRA group (Table 2).

**TABLE 2:** Hazard ratio of outcomes between non-steroidal MRA and steroidal-MRA groups.

Outcome	Non-steroidal MRA group (n = 1,378)	Steroidal MRA group (n = 1,378)	HR (95% CI)	*P* Value	E-value (95% LCL)
	Events (%)	Events (%)			
Primary outcome					
SICM	41 (3.0)	71 (5.2)	0.59 (0.40,0.87)	0.007	2.8 (1.6)
Secondary outcomes					
All-cause mortality	39 (2.8)	89 (6.5)	0.45 (0.31,0.65)	<0.001	3.9 (2.5)
Surrogate outcome	14 (1.0)	30 (2.2)	0.48 (0.25,0.90)	0.020	3.6 (1.5)

CI, confidence interval; HR, hazard ratio; LCL, lower confidence limit; SICM, Sepsis-induced cardiomyopathy.

**FIGURE 2:**
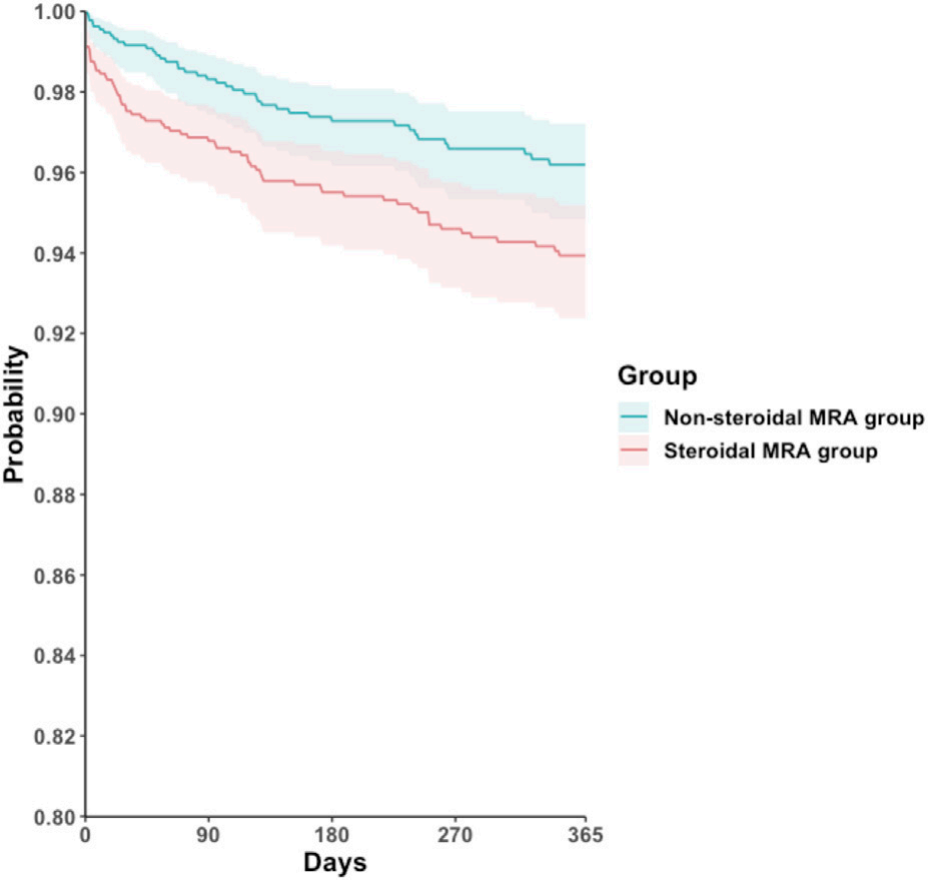
Kaplan-Meier time-to-event free curves of primary outcome comparison to non-steroidal MRA and steroidal-MRA groups. MRA, mineralocorticoid receptor antagonist.

For the primary outcome, SICM occurred in 41 patients (3.3%) in the non-steroidal MRA group and 71 patients (5.2%) in the steroidal MRA group. The HR was 0.59 (95% CI 0.40 to 0.87, p = 0.007), with an E value of 2.8. Kaplan–Meier curves further showed a consistently higher free probability in the non-steroidal MRA group compared with the steroidal MRA group (log-rank P = 0.007, Figure 2).

Regarding secondary outcomes, all-cause mortality was observed in 39 patients (2.8%) in the non-steroidal MRA group and 89 patients (6.5%) in the steroidal MRA group. The HR was 0.45 (95% CI 0.31 to 0.65, p < 0.001), and the E value was 3.9.

The surrogate outcome occurred in 14 patients (1.0%) in the non-steroidal MRA group and 30 patients (2.2%) in the steroidal MRA group. The HR for the surrogate outcome was 0.48 (95% CI 0.25 to 0.90, p = 0.020), with an E value of 3.6.

### Subgroup analysis

**FIGURE 3:**
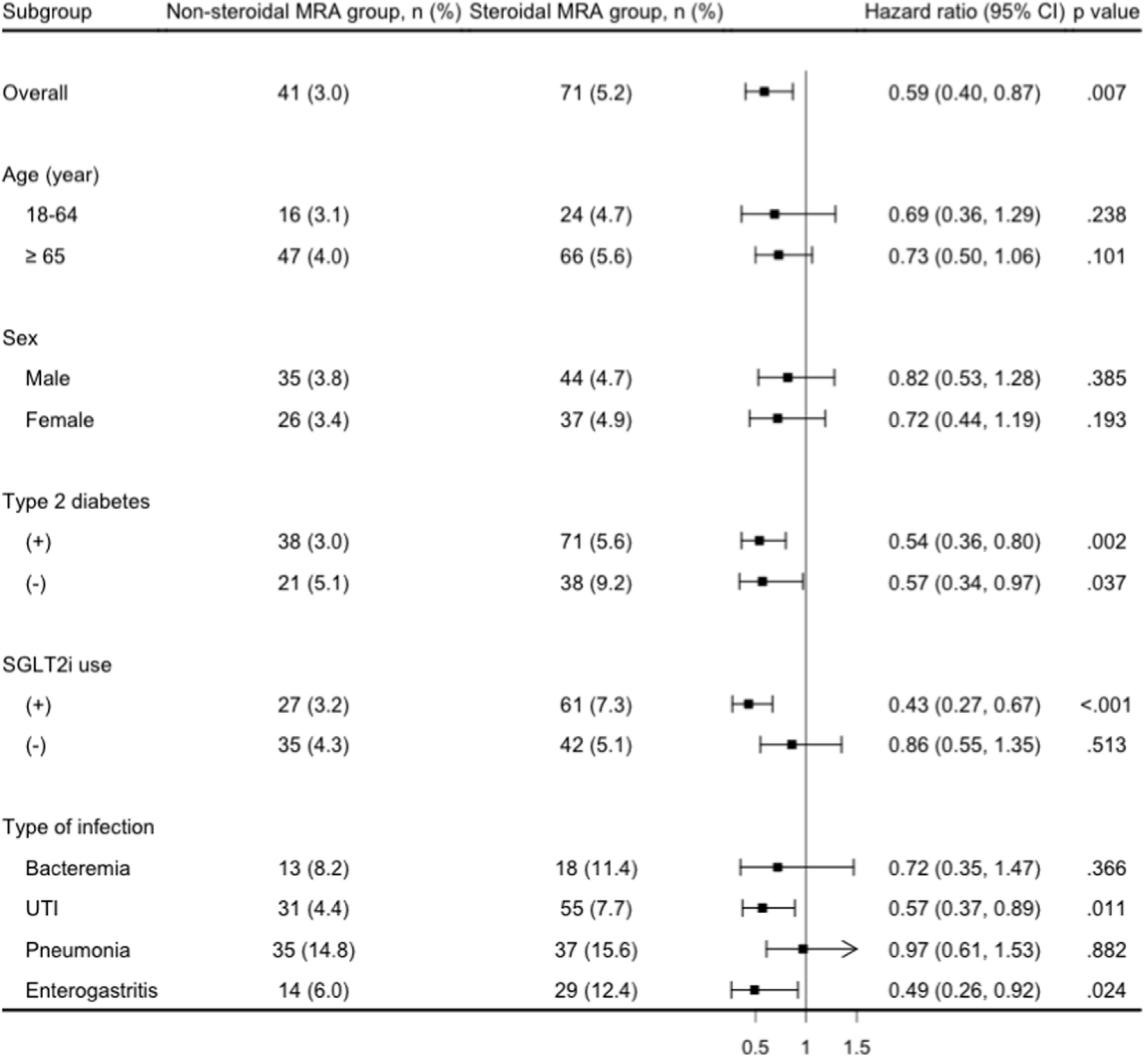
Subgroup analysis for the risk of primary outcome comparison to non-steroidal MRA and steroidal-MRA groups. CI, confidence interval; MRA, mineralocorticoid receptor antagonist; SGLT2i, sodium-glucose cotransporter 2 inhibitor; UTI, urinary tract infection.

Figure 3 shows the subgroup results for the association between non-steroidal MRA and the risk of SICM. In age stratified analyses, the HRs were 0.69 (95% CI 0.36 to 1.29, p = 0.238) among patients aged 18–64 years and 0.73 (95% CI 0.50 to 1.06, p = 0.101) among those aged 65 years or older. When stratified by sex, the HRs were 0.82 (95% CI 0.53 to 1.28, p = 0.385) in males and 0.72 (95% CI 0.44 to 1.19, p = 0.193) in females.

Among patients with type 2 diabetes, the HR was 0.54 (95% CI 0.36 to 0.80, p = 0.002), whereas among those without type 2 diabetes, the HR was 0.57 (95% CI 0.34 to 0.97, p = 0.037). In analyses stratified by sodium–glucose cotransporter-2 inhibitors (SGLT2is) use, the HR was 0.43 (95% CI 0.27 to 0.67, p < 0.001) among SGLT2i users and 0.86 (95% CI 0.55 to 1.35, p = 0.513) among non users. When stratified by type of infection, the HRs were 0.72 (95% CI 0.35 to 1.47, p = 0.366) for bacteremia, 0.57 (95% CI 0.37 to 0.89, p = 0.011) for urinary tract infection, 0.97 (95% CI 0.61 to 1.53, p = 0.882) for pneumonia, and 0.49 (95% CI 0.26 to 0.92, p = 0.024) for enterogastritis.

### Sensitivity analysis

To assess potential residual confounding, a negative control outcome that was not expected to be influenced by the exposure was examined. No association was identified between non-steroidal MRA use and the risk of skin cancer (HR 0.90 [95% CI 0.46–1.75], p = 0.756; Supplementary Table S4). Landmark analyses yielded results consistent with those of the primary analysis. During the follow up period from 1 day to 3 months, the HR was 0.55 (95% CI 0.36–0.86, p = 0.007), and during the follow up period from 1 day to 6 months, the HR was 0.63 (95% CI 0.44–0.93, p = 0.017; Supplementary Table S5). Regarding safety outcomes, no significant differences were observed between the non-steroidal MRA and steroidal MRA groups in the risks of hyperkalemia (HR 0.98, 95% CI 0.76–1.26; P = 0.870) or acute kidney injury (HR 0.94, 95% CI 0.78–1.13; P = 0.516) (Supplementary Table S6). In an additional analysis using a biomarker-defined myocardial injury, non-steroidal MRA use was associated with a significantly lower risk compared with steroidal MRA use (HR 0.38, 95% CI 0.19–0.77; P = 0.005) (Supplementary Table S8).

## Discussion

In this large real-world cohort of 2,756 patients with DKD, finerenone use was consistently associated with a lower risk of SICM compared with steroidal MRAs. Notably, this association was observed across multiple clinically relevant endpoints, including the primary outcome of SICM, surrogate markers of myocardial injury and dysfunction, and all-cause mortality within 1 year. Importantly, the association between finerenone use and reduced risk of SICM remained directionally consistent across most prespecified subgroup analyses. In addition, negative control outcome analyses and landmark analyses provided further support for the robustness of our findings. Given the heightened vulnerability of patients with DKD to sepsis-related cardiac complications, these findings address an important unmet clinical need in this high-risk population. Importantly, because SICM was ascertained using EHR-recorded diagnostic codes and structured LVEF data rather than systematic echocardiographic screening, our findings should be interpreted as associations with clinically recognized or EHR-recorded SICM, rather than the true echocardiography-defined incidence of SICM.

Although SGLT2i use was well balanced after PSM, the marked pre-matching difference in SGLT2i use deserves consideration. Patients treated with finerenone were substantially more likely to receive SGLT2i before matching, suggesting that they may have been managed in a more contemporary treatment era or by clinicians more likely to prescribe guideline-directed cardiorenal therapies. This pattern may reflect differences in patient selection, access to newer medications, clinical monitoring, and overall intensity of care. Therefore, the observed association between finerenone and lower SICM risk may partly reflect a broader “healthy user” or “intensive care” effect rather than the pharmacologic effect of finerenone alone.

Our findings extend prior evidence on the cardiovascular benefits of MRAs in patients with DKD into the setting of acute systemic infection. Large randomized trials such as FIDELIO-DKD and FIGARO-DKD demonstrated that finerenone reduces the risk of cardiovascular and renal events in patients with DKD (Pitt et al., 2021; Bakris et al., 2020); however, these trials were not designed to evaluate outcomes related to sepsis or sepsis-associated cardiac dysfunction. Likewise, the existing literature on SICM has predominantly focused on pathophysiology and prognostic implications, with limited evidence regarding pharmacological strategies aimed at preventing sepsis-related cardiac dysfunction (Sato et al., 2025; Boissier and Aissaoui, 2022).

Emerging real-world data have begun to suggest that certain cardiometabolic therapies may influence the risk of SICM. A recent propensity score–matched retrospective cohort study reported that baseline use of SGLT2i was associated with a lower incidence of SICM and improved cardiovascular outcomes among patients with diabetes and sepsis, compared with dipeptidyl peptidase-4 inhibitor therapy (Wu et al., 2025a). Although the pharmacologic mechanisms of SGLT2is differ from those of MRAs, these findings collectively support the concept that modulation of cardiometabolic and inflammatory pathways may affect susceptibility to sepsis-related myocardial dysfunction. Within this evolving evidence base, our study provides complementary real-world comparative data suggesting that non-steroidal MRAs with finerenone may be associated with a lower risk of SICM and related adverse outcomes compared with steroidal MRAs in patients with DKD. Importantly, our analysis addresses a distinct clinical question focused on the prevention of sepsis-related cardiac dysfunction, thereby extending existing heart failure and cardiometabolic literature into a previously underexplored high-risk context.

Although the present study was not designed to establish causality or directly interrogate biological mechanisms, several pathophysiological considerations may help contextualize our findings. SICM is thought to arise from a complex interplay of systemic inflammation, oxidative stress, endothelial dysfunction, and myocardial metabolic derangement, leading to transient but clinically consequential myocardial depression (Stanzani et al., 2019; Hollenberg and Singer, 2021). MR signaling has been implicated in multiple pathways that overlap with these processes, including promotion of inflammatory responses, vascular dysfunction, and myocardial vulnerability under stress conditions (Belden et al., 2017; Fiebeler and Luft, 2005; McCurley and Jaffe, 2012; Faulkner and Belin de Chantemèle, 2019). Additionally, experimental studies have shown that mineralocorticoid receptor activation can amplify inflammatory signaling pathways, including increased expression of proinflammatory cytokines such as interleukin-6 and tumor necrosis factor-α, which are also implicated in the pathogenesis of SICM (Thangaraj et al., 2022; Fan and Wu, 2024; Yang et al., 2025). In patients with DKD, chronic MR activation may further amplify these maladaptive responses, potentially increasing susceptibility to sepsis-related myocardial dysfunction. (Mende et al., 2023; Epstein et al., 2022).

Non-steroidal MRAs such as finerenone differ from conventional steroidal MRAs in their receptor selectivity and tissue distribution, allowing effective attenuation of pathogenic MR signaling with fewer off-target effects (Wu et al., 2025b; Kolkhof et al., 2017). In the context of sepsis, marked by acute inflammatory and hemodynamic stress, such pharmacological properties may theoretically translate into greater myocardial resilience (Kox et al., 2026). The observed consistency between reduced SICM risk, lower rates of biomarker-defined myocardial injury, and all-cause mortality in our study provides indirect support for this biological plausibility, while acknowledging that alternative explanations, including residual confounding, cannot be fully excluded. The substantial association observed for all-cause mortality should also be interpreted in a broader biological context. Although reduced clinically recognized SICM may partly contribute to improved survival, the mortality difference is unlikely to be explained by myocardial effects alone. MR activation has been implicated not only in myocardial injury but also in systemic inflammation, oxidative stress, endothelial dysfunction, vascular permeability, and kidney injury, all of which are relevant to the pathophysiology of sepsis (Nakamori et al., 2020). Compared with steroidal MRAs, finerenone is a more selective non-steroidal MRA with distinct tissue distribution and may theoretically modulate inflammatory and vascular pathways beyond the myocardium (Rahman et al., 2023). Therefore, the lower mortality observed in the finerenone group may reflect a combination of reduced cardiac vulnerability, improved cardiorenal stability, and broader attenuation of maladaptive inflammatory responses during acute infection. However, the observed association with all-cause mortality requires particularly cautious interpretation. The magnitude of the mortality association was larger than that observed for SICM and appears greater than the effect sizes reported in randomized trials of finerenone in patients with diabetic kidney disease. Therefore, this finding should not be interpreted as evidence that finerenone directly confers a large mortality benefit in the setting of sepsis. Although reduced clinically recognized SICM may partly contribute to improved survival, the mortality difference is unlikely to be fully explained by the proposed myocardial mechanism alone. Alternative explanations, including residual confounding, healthy-initiator or healthy-adherer bias, treatment-era effects, differences in clinical monitoring, and unmeasured differences in sepsis severity or overall care quality, may have contributed substantially. Thus, the mortality result should be regarded as a secondary, hypothesis-generating observation rather than definitive evidence of a causal treatment effect.

From a clinical perspective, our findings suggest that the choice of MRAs in patients with DKD may have implications beyond chronic cardiovascular and renal protection. Patients with DKD are at high risk for sepsis and sepsis-related cardiac complications, yet no pharmacological strategy has been prospectively validated for the prevention of SICM (Lai et al., 2013; Mansur et al., 2015). In this context, the observed association between finerenone use and a lower risk of SICM raises the possibility that non-steroidal MRAs may confer upstream cardioprotection in vulnerable patients before overt heart failure develops. Importantly, our study addresses a preventive paradigm distinct from heart failure treatment. While randomized trials such as FINEART-HF demonstrated that finerenone reduces heart failure–related events in patients with mildly reduced and preserved ejection fraction, those studies evaluated finerenone as a therapeutic intervention for established heart failure rather than for the prevention of SICM (Solomon et al., 2024). Nevertheless, the benefits observed in FINEART-HF support the concept that non-steroidal MRAs exerts cardioprotective effects in settings characterized by heightened inflammatory and hemodynamic stress (Ferreira et al., 2024; Kintscher and Edelmann, 2023). Taken together, these findings suggest that finerenone may represent a rational option in patients with DKD not only for long-term cardiorenal protection but also for mitigating the risk of acute, stress-related cardiac complications. However, prospective studies are needed before any changes to clinical practice can be recommended.

The racial distribution of the study population also requires consideration. Before matching, Asian patients were over-represented in the finerenone group, which may reflect regional differences in finerenone adoption, prescribing behavior, access to newer cardiorenal therapies, or healthcare-system characteristics within the TriNetX network. Although race was included in the propensity score model and was well balanced after matching, residual regional or site-level differences in clinical practice may still have influenced the observed associations.

### Limitations

This study has several limitations that should be acknowledged. First, the TriNetX platform is a registry-based electronic health record database, which may be subject to patient misclassification or underrepresentation, particularly among individuals with milder disease or limited engagement with healthcare services, potentially affecting the generalizability of our findings. In addition, the use of diagnostic and procedural codes to define exposures, covariates, and outcomes introduces the possibility of misclassification bias. To mitigate this concern, we conducted negative control outcome analyses using clinically unrelated conditions, which demonstrated null associations and thereby reduced the likelihood that systematic coding-related bias accounted for the observed results. Another important limitation is incomplete echocardiographic data. Although LVEF ≤50% was included as one component of the SICM definition, structured LVEF values are not uniformly available in TriNetX. The platform does not allow investigators to reliably distinguish between patients who did not undergo echocardiography, patients with LVEF documented only in unstructured reports, and patients with missing or unmapped echocardiographic data. Therefore, we were unable to quantify the completeness of LVEF measurements in the matched cohorts. Missing LVEF data may have led to underascertainment of SICM and may have affected the absolute incidence estimates. In addition, differential use of echocardiography, biomarker testing, or intensive monitoring between treatment groups could have introduced ascertainment bias. Accordingly, our outcome should be interpreted as clinically recognized or EHR-recorded SICM rather than the true echocardiography-defined incidence of SICM.

Second, medication exposure was defined using prescription records during the 12 months before the index diagnosis, and detailed data on dispensing, dosage, titration, treatment duration, discontinuation, and adherence were unavailable. Third, although extensive PSM was applied to balance a broad range of measured baseline characteristics, residual confounding from unmeasured or incompletely captured variables cannot be entirely excluded. Generalizability may also be limited by differences in racial composition and potential regional or site-level clustering. Before matching, Asian patients were over-represented in the finerenone group, suggesting possible differences in regional prescribing patterns, healthcare access, or adoption of newer cardiorenal therapies. Although race was balanced after PSM, the TriNetX platform provides de-identified, federated, aggregated data and does not allow investigators to reliably evaluate hospital-level clustering, regional variation, or site-specific differences in sepsis management, echocardiographic testing, and follow-up intensity. Therefore, residual confounding related to healthcare system, region, or site-level practice patterns cannot be excluded, and the findings may not be fully generalizable to populations or healthcare systems with different racial composition, treatment availability, or clinical practice patterns. To further evaluate the potential influence of unmeasured confounding, we calculated E-values, which indicated that a substantial degree of unmeasured confounding would be required to fully explain the observed associations, supporting the robustness of our findings. Fourth, healthy user bias and treatment-era effects cannot be fully excluded. Before matching, SGLT2 inhibitor use was substantially higher in the finerenone group than in the steroidal MRA group, which may indicate that finerenone users were more likely to receive contemporary guideline-directed cardiorenal therapies, closer clinical follow-up, and more intensive overall care. Although SGLT2 inhibitor use and other measured covariates were balanced after PSM, residual differences in healthcare access, physician prescribing behavior, monitoring intensity, socioeconomic status, and unmeasured disease-management quality may have persisted. Therefore, the present findings should be interpreted as hypothesis-generating and not as evidence of a direct causal protective effect of finerenone. Fifth, because the index event was defined using broad infectious disease codes rather than validated sepsis-specific codes, the cohort may have included patients with heterogeneous infection severity. Therefore, the findings should be interpreted as clinically recognized cardiac dysfunction following documented infection rather than as definitive evidence specific to validated sepsis.

Finally, the observed reduction in all-cause mortality was substantial and disproportionate to the reduction observed for SICM, and should therefore be interpreted cautiously. Cause-specific mortality information was not available within the database, preventing distinction between cardiovascular and non-cardiovascular deaths. Moreover, sepsis severity scores, vasopressor use, intensive care parameters, inflammatory biomarkers, medication adherence, and detailed treatment processes were unavailable. As a result, we could not determine whether the mortality difference was related to reduced clinically recognized SICM, broader anti-inflammatory or cardiorenal effects, or residual differences in baseline health status and overall care quality. In addition, all-cause mortality may have acted as a competing event for SICM, as patients with severe sepsis who died early may not have survived long enough to develop or be diagnosed with clinically recognized SICM. Because competing-risk methods, such as the Fine–Gray subdistribution hazard model, are not currently supported by the TriNetX platform, deaths were treated as censoring events in the present analysis.

## Conclusion

In this real-world cohort of patients with DKD and sepsis, finerenone use was associated with a lower observed risk of clinically recognized SICM compared with steroidal MRAs. Although lower all-cause mortality was also observed, the magnitude of this association was substantial and should not be interpreted as definitive evidence of a causal mortality benefit. Residual confounding, healthy-initiator or healthy-adherer bias, treatment-era effects, and unmeasured differences in sepsis severity or care intensity may have contributed. These findings should therefore be considered hypothesis-generating and require confirmation in prospective studies with systematic cardiac imaging, detailed sepsis severity assessment, and adjudicated cause-specific outcomes.

## Data Availability

The data analyzed in this study is subject to the following licenses/restrictions: The datasets generated and analyzed during this study are not publicly available. Data were obtained from the TriNetX research network and are available only in de-identified, aggregated form. Access to the data is restricted and requires application to TriNetX, subject to institutional agreements and compliance with data privacy regulations. Requests to access these datasets should be directed to https://trinetx.com/.
