# Supplementary material for: Comparative effectiveness of finerenone versus steroidal mineralocorticoid receptor antagonists on sepsis-induced cardiomyopathy in patients with diabetic kidney disease: a real-world cohort study
Source: Front Pharmacol. 2026 Jul 28;17:1837944. doi: 10.3389/fphar.2026.1837944 (PMC13457483; doi:10.3389/fphar.2026.1837944)
Supplement: Supplementary file 1 [file Table1.docx]

**Table S1.** Demographic, diagnostic, procedural, medication, visit, and laboratory codes utilized in the definition of the cohorts.

| **Category** | **Code** | **Description** |
| --- | --- | --- |
| **Index drug** | | |
| medication | NLM:RXNORM:2562811 | finerenone |
| medication | NLM:RXNORM:9997 | spironolactone |
| medication | NLM:RXNORM:298869 | eplerenone |
| **Infection** | | |
| diagnosis | UMLS:ICD10CM:A00-B99 | Certain infectious and parasitic diseases |
| **DKD** |  |  |
| diagnosis | UMLS:ICD10CM:N18 | Chronic kidney disease (CKD) |
| diagnosis | UMLS:ICD10CM:R80 | Proteinuria |
| laboratory | UMLS:LNC:9318-7 | Albumin/Creatinine [Mass Ratio] in Urine (at least 30.00 mg/g{creat}) |
| diagnosis | UMLS:ICD10CM:E11 | Type 2 diabetes mellitus |

**Table S2.** Definitions of covariates coding used in this study.

| **Code** | **Description** |
| --- | --- |
| AI | Age at Index |
| 2106-3 | White |
| F | Female |
| 2054-5 | Black or African American |
| M | Male |
| 2028-9 | Asian |
| A41 | Other sepsis |
| E11.2 | Type 2 diabetes mellitus with kidney complications |
| E11.3 | Type 2 diabetes mellitus with ophthalmic complications |
| E11.4 | Type 2 diabetes mellitus with neurological complications |
| E11.5 | Type 2 diabetes mellitus with circulatory complications |
| E78 | Disorders of lipoprotein metabolism and other lipidemias |
| I48 | Atrial fibrillation and flutter |
| F17 | Nicotine dependence |
| E66 | Overweight and obesity |
| CV100 | BETA BLOCKERS/RELATED |
| A10BA | Biguanides |
| A10BK | Sodium-glucose co-transporter 2 (SGLT2) inhibitors |
| CV800 | ACE INHIBITORS |
| CV805 | ANGIOTENSIN II INHIBITOR |
| 9068 | Lactate [Moles/volume] in Serum, Plasma or Blood |
| 9063 | C reactive protein [Mass/volume] in Serum, Plasma or Blood |
| 9002 | Cholesterol in LDL [Mass/volume] in Serum or Plasma |
| 9037 | Hemoglobin A1c/Hemoglobin.total in Blood |
| 8001 | Glomerular filtration rate/1.73 sq M.predicted [Volume Rate/Area] in Serum, Plasma or Blood by Creatinine-based formula (MDRD) |
| 9083 | BMI |

**Table S3.** Definitions of outcomes coding used in this study.

| **Code** | **Description** |
| --- | --- |
| **SICM** | |
| UMLS:ICD10CM:J81 | Pulmonary edema |
| UMLS:ICD10CM:I50 | Heart failure |
| UMLS:ICD10CM:R57.0 | Cardiogenic shock |
| TNX:FINDING:2003 | Left Ventricular Ejection Fraction (LVEF) (%) (at most 50.00 % (most recent occurrence)) |
| **All-cause mortality** | |
| Deceased | Deceased |
| UMLS:ICD10CM:R99 | Ill-defined and unknown cause of mortality |
| **Surrogate outcome** | |
| UMLS:LNC:33762-6 | Natriuretic peptide.B prohormone N-Terminal [Mass/volume] in Serum or Plasma (at least 1800.00 pg/mL (most recent occurrence)) |
| TNX:9005 | Troponin I.cardiac [Mass/volume] in Serum, Plasma or Blood (at least 0.50 ng/mL (most recent occurrence)) |
| TNX:FINDING:2003 | Left Ventricular Ejection Fraction (LVEF) (%) (at most 50.00 % (most recent occurrence)) |

**Table S4**. Negative outcome control.

| Outcome | HR (95% CI) | *P* value |
| --- | --- | --- |
|  |  |  |
| Skin cancer | 0.90 (0.46,1.75) | 0.756 |

**Table S5**. Sensitivity analysis with landmark analysis of the primary outcome.

| Outcome | HR (95% CI) | P value |
| --- | --- | --- |
| 1-day to 3-months | 0.55 (0.36,0.86) | 0.007 |
| 1-day to 6-months | 0.63 (0.44,0.93) | 0.017 |

**Table S6**. Safety outcomes after sepsis.

| Outcome | HR (95% CI) | P value |
| --- | --- | --- |
| Hyperkalemia | 0.98 (0.76,1.26) | 0.870 |
| Acute kidney injury | 0.94 (0.78,1.13) | 0.516 |

**Table S7**. Absolute event numbers and incidence rates for the primary and secondary outcomes.

| Outcome | Non-steroidal MRA group (n = 1,378)  Events (Incidence rate per 100 person-years) | Steroidal MRA group (n = 1,378)  Events (Incidence rate per 100 person-years) |
| --- | --- | --- |
| Primary outcome |  |  |
| SICM | 41 (3.0) | 71 (5.2) |
| Secondary outcomes |  |  |
| All-cause mortality | 39 (2.8) | 89 (6.5) |
| Surrogate outcome | 14 (1.0) | 30 (2.2) |

**Table S8**. Additional analysis using biomarker-defined myocardial injury.

| Outcome | HR (95% CI) | P value |
| --- | --- | --- |
| Biomarker-defined cardiac injury | 0.38 (0.19,0.77) | 0.005 |
